# Dynamics of influenza in tropical Africa: Temperature, humidity, and co‐circulating (sub)types

**DOI:** 10.1111/irv.12556

**Published:** 2018-04-17

**Authors:** Wan Yang, Matthew J. Cummings, Barnabas Bakamutumaho, John Kayiwa, Nicholas Owor, Barbara Namagambo, Timothy Byaruhanga, Julius J. Lutwama, Max R. O'Donnell, Jeffrey Shaman

**Affiliations:** ^1^ Department of Environmental Health Sciences Columbia University New York NY USA; ^2^ Division of Pulmonary, Allergy, and Critical Care Medicine Columbia University New York NY USA; ^3^ National Influenza Center Uganda Virus Research Institute Entebbe Uganda; ^4^ Department of Epidemiology Columbia University New York NY USA

**Keywords:** Africa, humidity, influenza, intertypic interactions, temperature, tropical climate, Uganda

## Abstract

**Background:**

The association of influenza with meteorological variables in tropical climates remains controversial. Here, we investigate the impact of weather conditions on influenza in the tropics and factors that may contribute to this uncertainty.

**Methods:**

We computed the monthly viral positive rate for each of the 3 circulating influenza (sub)types (ie, A/H1N1, A/H3N2, and B) among patients presenting with influenza‐like illness (ILI) or severe acute respiratory infections (SARI) in 2 Ugandan cities (Entebbe and Kampala). Using this measure as a proxy for influenza activity, we applied regression models to examine the impact of temperature, relative humidity, absolute humidity, and precipitation, as well as interactions among the 3 influenza viruses on the epidemic dynamics of each influenza (sub)type. A full analysis including all 4 weather variables was done for Entebbe during 2007‐2015, and a partial analysis including only temperature and precipitation was done for both cities during 2008‐2014.

**Results:**

For Entebbe, the associations with weather variables differed by influenza (sub)type; with adjustment for viral interactions, the models showed that precipitation and temperature were negatively correlated with A/H1N1 activity, but not for A/H3N2 or B. A mutually negative association between A/H3N2 and B activity was identified in both Entebbe and Kampala.

**Conclusion:**

Our findings suggest that key interactions exist among influenza (sub)types at the population level in the tropics and that such interactions can modify the association of influenza activity with weather variables. Studies of the relationship between influenza and weather conditions should therefore determine and account for co‐circulating influenza (sub)types.

## 1 BACKGROUND

Influenza epidemics cause substantial morbidity and mortality worldwide. Weather conditions, such as humidity and temperature, have been identified as key factors shaping the dynamics of influenza transmission. In temperate regions, lower humidity and lower temperature have repeatedly been shown to be associated with the wintertime epidemics.^1^, ^2^, ^3^, ^4^, ^5^ In tropical regions, however, few studies have been conducted and, from the limited number of studies, findings on the impact of weather conditions are inconsistent. While some studies reported increased influenza circulation during the rainy seasons^5^, ^6^, ^7^, ^8^, ^9^, ^10^, ^11^, ^12^ and association with higher humidity and/or precipitation in the tropics,^5^, ^7^, ^13^ others reported no or contradicting effects of these climate variables.^14^, ^15^, ^16^, ^17^ Here, we investigate the relationship between weather conditions and influenza transmission in Uganda, a tropical country, as well as factors that may contribute to the aforementioned inconclusiveness.

Uganda is located between latitudes 4°N and 2°S on the East African Plateau, with a tropical climate. To enhance influenza surveillance in the country, the Uganda Virus Research Institute (UVRI) initiated a sentinel surveillance network in 2007 to monitor influenza‐like illness (ILI) and severe acute respiratory infections (SARI) nationwide. At the sentinel sites, respiratory specimens were collected from all ILI/SARI cases and tested for influenza viruses by (sub)type‐specific RT‐PCR. In this study, we utilized these surveillance data to study the transmission dynamics of influenza in 2 major Ugandan cities, Kampala and Entebbe, during 2007‐2015.

We used regression models to test the impact of temperature, humidity, and precipitation on influenza transmission. The two studied cities are in close vicinity with similar weather conditions. Comparing findings for these 2 cities thus allowed identification of inconsistencies and spurious associations. In addition, as the influenza samples were fully subtyped, we were able to address 3 questions that have been rarely, if ever, investigated: (i) How do effects of weather conditions vary by influenza (sub)type? (ii) How do interactions among influenza (sub)types manifest at the population level? And (iii) how do these viral interactions along with weather conditions shape the epidemic dynamics of each influenza virus?

## 2 METHODS

### 2.1 Ethics statement

In the context of routine public health surveillance, verbal consent was obtained from suspected cases ≥18 years of age and from parents or legal guardians for cases <18 years of age. The study was approved by the Research Ethics Committee at UVRI, the Uganda National Council for Science and Technology, and the Institutional Review Board at Columbia University Medical Center. The study's funders had no role in study design, data analysis and interpretation, manuscript preparation, or decision to publish.

### 2.2 Data

At each sentinel site, clinicians identified patients with influenza‐like illness (ILI) and severe acute respiratory infection (SARI) using an established protocol.^9^, ^10^ Per standard World Health Organization guidelines, patients met the case definition for ILI if they were ≥2 months of age, presenting with a fever (>38°C) and either cough or sore throat. SARI was defined as: (i) a child aged 2 months to <5 years requiring hospitalization, with recent onset of cough or difficulty breathing within 10 days of symptom onset and an additional indicator of respiratory distress; or (ii) a patient aged ≥5 years requiring hospitalization, with a history of fever presenting with cough, shortness of breath, or difficulty breathing within 10 days of symptom onset.^9^, ^10^ Naso‐ and/or oropharyngeal swab samples were collected at enrollment from ILI/SARI patients, and all samples were tested for influenza viruses by (sub)type‐specific RT‐PCR using primers provided by the U.S. Centers for Disease Control and Prevention.^10^ If specimens were positive for influenza A, further subtyping was done for seasonal A/H1N1, A/H3N2, A/H5, and the 2009 pandemic influenza A/H1N1 (A/pdmH1).

Data for Kampala were compiled from 6 sentinel sites located in the city (ie, IHK/Surgery, Kibuli, Kisenyi, Kiswa, Kitebi, and Nsambya), and those for Entebbe were compiled from 1 sentinel site. The sentinel site in Entebbe reported regularly from 2007 to 2015. For Kampala, while at least 1 site was reporting at any time during 2008‐2015, the number of reporting sites fluctuated over time. To control for this fluctuation in reporting rate, we used the proportions of specimens testing positive for A/H1N1, A/H3N2, or B, respectively, among all ILI/SARI specimens as proxies for activity of the 3 influenza viruses. Due to the low numbers recorded, we aggregated the data to monthly intervals.

For Entebbe, monthly precipitation, mean temperature, and relative humidity data from January 2007 to December 2015 were compiled from 2 sources. Specifically, monthly precipitation values were compiled from Uganda Bureau of Statistics (UBoS)'s annual statistical abstract.^18^ Daily mean temperature and relative humidity (RH) recorded at Entebbe International Airport were obtained from Weather Underground.^19^ Specific humidity, a form of absolute humidity (AH), was calculated from RH and temperature using the Clausius‐Clapeyron equation.^20^ Monthly averages of temperature, RH, and AH were then computed from these daily records. We used this combined dataset for Entebbe for the full statistical analysis (2.3). For Kampala, however, similar ground station data were only partially available for precipitation (2008‐2015), maximum and minimum temperature (2008‐2014), and RH (2008 and 2009), all at monthly intervals, from UBoS annual statistical abstracts.^18^ These weather variables were also available for Entebbe, from the same UBoS reports. We therefore used the average of the maximum and minimum temperature for each month to represent the monthly mean temperature and used these values along with monthly precipitation in the partial statistical analysis for a comparison of the 2 cities (2.4).

### 2.3 Full statistical analysis

As complete data for temperature, precipitation, relative and absolute humidity are available for Entebbe, we performed a statistical analysis of the relationship between influenza activity and these 4 weather variables for the city during January 2007‐December 2015. We refer to this analysis as the full statistical analysis hereafter. We first examined the relationship for each influenza (sub)type and all strains combined without adjustment for co‐circulating influenza (sub)types (2.3.1), as has been done in previous studies. In the second part of this analysis, we reexamined the relationship with adjustment for co‐circulating influenza (sub)types (2.3.2). For simplicity, we combined all A/H1N1 cases, including those caused by the pre‐2009 seasonal strain and those by the 2009 pandemic strain. To control for factors related to the 2009 pandemic (eg, higher population susceptibility), we excluded the period of July‐December 2009 in all analyses [ie, for A/H1N1 as well as other (sub)types and all strains combined]. In addition, to reduce biases due to small sample size, months with <5 ILI/SARI specimens (15 out of 102 months) were excluded in the models. For all models, all predictor variables were normalized to have zero mean and unit variance.

#### 2.3.1 Influenza activity and weather conditions

We used logistic regression models^21^ with 1 autoregressive term to examine the impact of weather variables on monthly influenza activity. The basic model for this analysis took the following form:

(1) $$\begin{array}{cc} & \text{Logit}\;\left(Flu\right)∼\beta_{0}+\beta_{1}\left(Flu\;at\;lag\text{‐}\;\;1\right)\\ & +\;\beta_{2-12}\;Seasonality\left(t\right)+\beta_{13}\left(Temp\right)+\beta_{14}\left(HUM\right) \end{array}$$

where Logit (*p*) is the logit function, that is, ln[*p*/(1‐*p*)]; *Flu* represents the monthly viral positive rate for either A/H1N1, A/H3N2, B, or all influenza viruses combined (All); *Flu at lag‐1* is the lag‐1 autoregressive term*. Seasonality* (*t*) is a function to account for seasonality in the data; here, we used harmonics of the form $\sum_{k=1}^{6}\left\{a_{k}\cos\left[\left(2\pi k/12\right)t\right]+b_{k}\sin\left[\left(2\pi k/12\right)t\right]\right\}$ with *t* as the month index (eg, *t *=* *1 for January and *t *=* *2 for February).^22^ (Note that as $sin\left[\left(\frac{2\pi k}{12}\right)t\right]$ for *k *=* *6 is always 0, there are 11 coefficients, ie, β_*2‐12*_, in this formula.) *Temp* represents temperature. *HUM* represents 1 of the 3 humidity‐related variables (ie, precipitation, RH, and AH). That is, only 1 humidity‐related variable was included in the model in order to avoid over fitting (same for all other models in this study). For models that include both temperature and a humidity‐related variable, we did not adjust for collinearity between the 2 terms, since this effect was not severe (the variance inflation factors^23^ were <5 for all variable pairs).

#### 2.3.2 Influenza activity, weather conditions, and co‐circulating (sub)types

**Figure 1:**
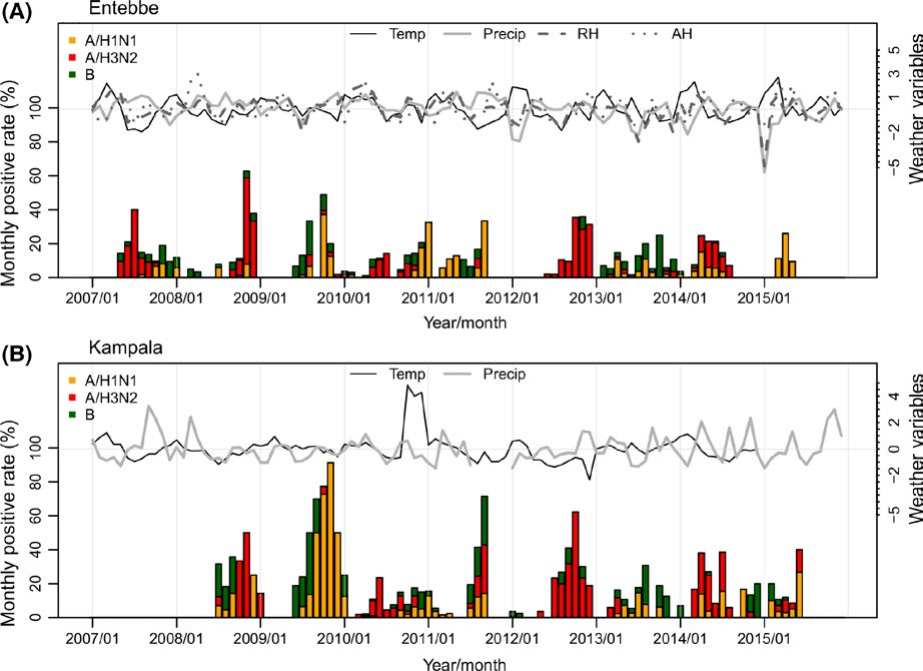
Monthly viral positive rates for different influenza viruses (*y*‐axis on the left) and weather variables (*y*‐axis on the right) in Entebbe and Kampala. Weather variables are standardized to have zero mean and unit variance. Humidity data (RH and AH) are not available for Kampala

To examine the potential confounding and modification effect of co‐circulating (sub)types on the relationship between influenza activity and weather conditions, we also developed models that adjusted for activity of co‐circulating (sub)types. The basic model for this analysis assumed the following structure:

(2) $$\begin{array}{cc} & \text{Logit}\;\left(Flu\right)∼\beta_{0}+\beta_{1}\left(Flu\;at\;lag\text{‐}\;\;1\right)+\beta_{2-12}\;Seasonality\left(t\right)\\ & +\;\beta_{13}\left(Temp\right)+\beta_{14}\left(HUM\right)+\beta_{15}\;\left(co\text{‐}\;\;Flu1\right)+\beta_{16}\left(co\text{‐}\;\;Flu2\right) \end{array}$$

where *co‐Flu1* and *co‐Flu2* represent concurrent viral positive rates for the other 2 co‐circulating (sub)types. For instance, for A/H1N1 (ie, *Flu*=A/H1N1), *co‐Flu1* and *co‐Flu2* are A/H3N2 and B, respectively. Note that, the 3 *Flu* time series (ie, proportions of specimens testing positive for A/H1N1, A/H3N2, or B, respectively, among all ILI/SARI visits) are *not* dependent on one another due to sharing the same denominator, as the denominator is not the number of influenza positive visits and the 3 proportions at each time step do not sum to 100% (Figure 1). Other variable settings were the same as in Equation (1).

#### 2.3.3 Best‐fit models

To thoroughly search for the best‐fit model for each influenza time series, we tested all possible combinations of *co‐Flu1*,* co‐Flu2*, temperature, and a humidity‐related variable. Note this test also included models without any co‐Flu variable or weather variable. We then pooled all models and selected the one with the lowest Bayesian information criterion (BIC)^24^ as the best‐fit model for each influenza time series.

#### 2.3.4 Leave‐one‐out cross‐validation

We performed cross‐validation of the 2 basic models (Equations (1) and (2)), by running the models excluding data from one of the study years (ie, 2007‐2015). For instance, in a leave‐one‐out cross‐validation for Year 2008, data for 2008 were excluded and the remainder used to fit the models. Model estimates for each covariate across years were then compared.

### 2.4 Partial statistical analysis

Statistical correlations consistently identified in multiple locations would strengthen these inferred associations. Due to a lack of humidity data for Kampala, we were unable to perform a full analysis as performed for Entebbe (2.3) and compare results between the 2 locations. Nevertheless, as a partial validation, we fitted the 2.3.1 and 2.3.2 models for both Kampala and Entebbe using a subset of weather variables (ie, temperature and precipitation), for which data were available for both cities for at least 3 years. That is, only precipitation was used as an *HUM* variable in Equations (1) and (2). For this comparison, we restricted the study period to July 2008‐December 2014 when data for influenza activity, temperature, and precipitation were available for both cities. In addition, to control for effects due to the 2009 pandemic, we excluded the 2009 pandemic (July‐December 2009) as in the full analysis. As in 2.3, we pooled all models [ie, with or without adjustment for co‐circulating influenza (sub)types] and selected the one with the lowest BIC as the best‐fit model for each influenza time series. In addition, cross‐validation was performed for both cities as described in 2.3.4.

### 2.5 Software

All statistical analyses were performed in R language (R Foundation for Statistical Computing, Vienna, Austria). All logistic models were fitted using the “bayesglm” function in the “arm” package in R.^25^ The “bayesglm” method, developed by Gelman et al,^26^ takes a Bayesian approach to obtain stable logistic regression coefficients using weakly informative priors (the default prior is a Cauchy distribution).

## 3 RESULTS

### 3.1 Summary statistics

The surveillance network detected 514 influenza cases among 4477 ILI/SARI visits (11.48%) in Entebbe during January 2007‐December 2015 and 390 influenza cases among 2566 ILI/SARI visits (15.20%) in Kampala during July 2008‐December 2014 (Table 1). Among the confirmed influenza cases, the majority were infected by A/H3N2 (47.67% in Entebbe and 43.33% in Kampala). The 2009 A/H1N1 pandemic strain (A/pdmH1) was first detected in late June or early July 2009 in the 2 cities and quickly replaced the seasonal A/H1N1 strain. As such, A/pdmH1 accounted for the majority of A/H1N1 cases during the study period in both cities (Table 1). While the viral positive rate varied substantially by month, influenza epidemics recurred in all study years (Figure 1).

**Table 1:** Monthly influenza cases and weather conditions in Entebbe (January 2007‐December 2015) and Kampala (July 2008‐December 2014)

	Entebbe		Kampala	
	Total No. (%)^a^	Mean (SD)^b^	Total No. (%)	Mean (SD)
A/H1N1	26 (5.06%)	3.09% (6.91%)	20 (5.13%)	6.52% (15.43%)
A/pdmH1	126 (24.51%)	2.62% (6.69%)	95 (24.36%)	5.24% (13.71%)
A/H3N2	245 (47.67%)	4.07% (9.03%)	169 (43.33%)	6.59% (11.98%)
B	115 (22.37%)	2.36% (4.09%)	105 (26.92%)	4.79% (7.88%)
Coinfection (A/H3, pdmH1)	1 (0.19%)	‐	‐	
A/unsubtypeable	1 (0.19%)	‐	1 (0.26%)	‐
All flu	514	9.53% (12.14%)	390	17.95% (20.42%)
ILI/SARI visits	4477	41 (30)	2566	33 (25)
Temperature (°C)^c^: all months	‐	22.34 (0.54)	‐	22.81 (1.18)
Temperature (°C): dry seasons^d^	‐	22.31 (0.59)	‐	22.7 (1.13)
Temperature (°C): rainy seasons^e^	‐	22.37 (0.49)	‐	22.92 (1.23)
Precipitation (mm/mo): all months	‐	126.5 (84.29)	‐	99.65 (67.88)
Precipitation (mm/mo): dry seasons	‐	80.63 (56.03)	‐	70.06 (55.4)
Precipitation (mm/mo): rainy seasons	‐	172.38 (83.17)	‐	129.24 (66.86)
RH (%): all months	‐	79.6 (2.97)	‐	‐
RH (%): dry seasons	‐	78.62 (3.52)	‐	‐
RH (%): rainy seasons	‐	80.59 (1.86)	‐	‐
AH (g/kg): all months	‐	13.47 (0.47)	‐	‐
AH (g/kg): dry seasons	‐	13.27 (0.51)	‐	‐
AH (g/kg): rainy seasons	‐	13.67 (0.32)	‐	‐

a The denominator for the percentages is the total number of positive influenza samples;

b For the rows related to influenza viruses, the mean is the mean monthly percentage of specimens testing positive for a given influenza virus among all ILI/SARI visits in the same month;

c Temperature is the monthly mean for Entebbe and the average of the monthly maximum and minimum for Kampala (see main text for detail);

d Dry seasons are December‐February and June‐August;

e Rainy seasons are March‐May and September‐November.

Kampala and Entebbe are only 37 km apart, and hence, weather conditions are similar in the 2 cities (Figure S1). In both cities, temperature is high year‐round; mean temperature during the study period was above 20°C in both cities (Table 1 and Figure S1). Typically, each year has 2 rainy seasons (March‐May and September‐November; Figure 1 and Figure S1), with much higher levels of precipitation during rainy seasons (Table 1). In Entebbe, where humidity data were available, ambient humidity is high year‐round; monthly averages were always above 60% for RH and above 11 g/kg for AH during the study period and were slightly higher during rainy seasons (Table 1 and Figure S1).

### 3.2 Full statistical analysis for Entebbe

#### 3.2.1 Influenza activity and weather conditions, without adjusting for co‐circulating (sub)types

**Figure 2:**
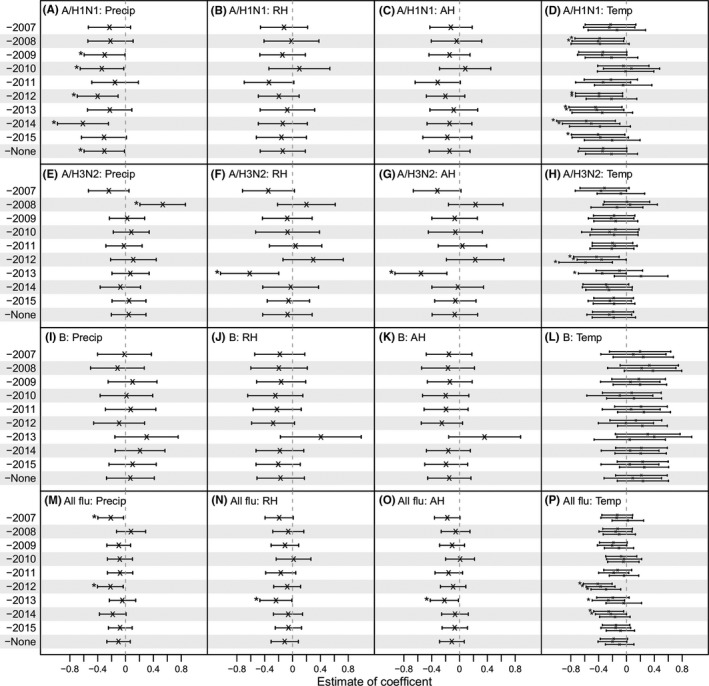
Estimated association of influenza epidemics with weather variables in Entebbe during 2007‐2015, without adjusting for co‐circulating (sub)types. Model coefficients estimated in the leave‐one‐out cross‐validation and over the entire study period are shown for A/H1N1 (1st row: A‐D), A/H3N2 (2nd row: E‐H), B (3rd row: I‐L), and all influenza viruses combined (4th row: M‐P). The year left out in the cross‐validation is shown on the *y*‐axis (eg, “‐2007” indicates data for Year 2007 were excluded, and “‐none” indicates data for the entire study period were included). The associations with precipitation (Precip), relative humidity (RH), absolution humidity (AH), and temperature (Temp) are shown in columns 1‐4; the vertical segments show the 95% confidence intervals for each variable; “x”s denote the mean and “*”s indicate variables significant at the 5% level. For temperature (last column), 3 estimates are shown for each dataset, corresponding to the 3 models using 1 of the 3 humidity variables (ie, precipitation, RH, and AH)

We first modeled the relationship between influenza activity and all 4 weather variables (ie, temperature, precipitation, relative, and absolute humidity) without adjusting for co‐circulating (sub)types, as commonly done in the previous studies. Over the entire study period (ie, 2007‐2015 excluding July‐December 2009 due to the pandemic), A/H1N1 activity correlated negatively with both precipitation and temperature (Figure 2A, D). Lower precipitation was significantly associated with higher A/H1N1 activity, with an odds ratio (OR) of 0.74 [0.55, 0.99] (mean and 95% confidence interval; *P* = .039); in the same model, lower temperature was also marginally significantly associated with higher A/H1N1 activity (OR: 0.71 [0.51, 1.00]; *P* = .053). In addition, estimated effects for these 2 weather variables during cross‐validation, where each test excluded 1 year of data, were in general in agreement (Figure 2A, D). Neither relative nor absolute humidity was identified as a significant correlate for A/H1N1 activity (Figure 2B for RH and 2C for AH).

Unlike A/H1N1, A/H3N2 and B activities were not significantly associated with any of the 4 weather variables during 2007‐2015. For A/H3N2, several exceptions were seen in the cross‐validation fits, including a positive correlation with precipitation when data for Year 2008 were excluded (Figure 2E), a negative correlation with RH and AH when data for Year 2013 were excluded (Figure 2F, G), and a number of negative correlations with temperature (Figure 2H). Overall, although not statistically significant, A/H3N2 activity appeared to be negatively correlated with temperature, with a mean OR, averaged over 30 models, of 0.82 (Figure 2H). Conversely, a positive correlation with temperature, although not statistically significant, was consistently estimated for influenza B; the mean OR was 1.19 across 30 models (Figure 2L).

For all influenza viruses combined, the models did not identify any significant correlates (Figure 2M‐P). The estimated OR was 0.90 [0.76, 1.07] (*P* = .24) for precipitation, 0.89 [0.73, 1.08] (*P* = .26) for RH, 0.90 [0.75, 1.07] (*P* = .23) for AH, and 0.85 [0.70, 1.04] (*P* > .05 for all 3 models) for temperature.

#### 3.2.2 Influenza activity, weather conditions, and co‐circulating (sub)types

**Figure 3:**
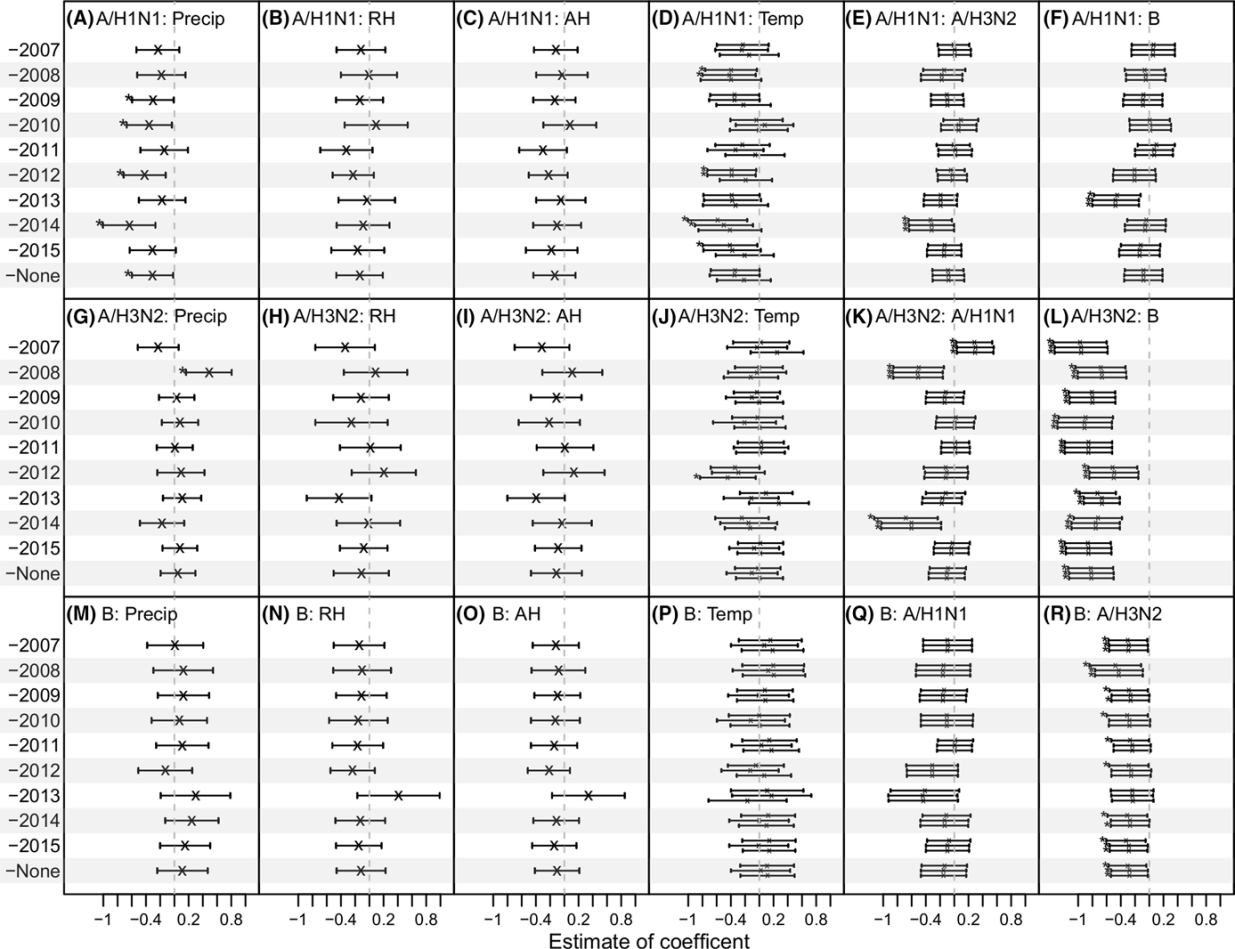
Estimated inter‐(sub)type interactions and association of influenza epidemics with weather variables adjusted for co‐circulating (sub)types in Entebbe during 2007‐2015. Model coefficients estimated in the leave‐one‐out cross‐validation and over the entire study period are shown for A/H1N1 (1st row: A‐F), A/H3N2 (2nd row: G‐L), and B (3rd row: M‐R). The year left out in the cross‐validation is shown on the *y*‐axis (eg, “‐2007” indicates data for Year 2007 were excluded, and “‐none” indicates data for the entire study period were included). The associations with precipitation (Precip), relative humidity (RH), absolution humidity (AH), and temperature (Temp) are shown in columns 1 to 4, and (sub)type interactions are shown in columns 5 and 6; the vertical segments show the 95% confidence intervals for each variable; “x”s denote the mean, and “*”s indicate variables significant at the 5% level. For temperature (4th column), 3 estimates are shown for each dataset, corresponding to the 3 models using 1 of the 3 humidity variables (ie, precipitation, RH, and AH)

In our second analysis, we further examined potential interactions among co‐circulating influenza (sub)types and the resulting impact on the relationship of epidemic activity with weather variables. Consistent with our first analysis, in models including 2 co‐circulating (sub)types along with both weather predictors (Equation (2)), A/H1N1 activity correlated negatively with both precipitation and temperature with nearly identical estimated effects (Figure 3A, D, and Figure S2). In addition, the estimated effects for all 4 weather variables, by both the models over the entire study period and leave‐one‐out cross‐validation, were nearly the same as estimated by the corresponding models without adjusting for co‐circulating (sub)types (Figure S2 A‐F). The correlation between A/H1N1 activity and concurrent A/H3N2 activity varied slightly by included data (as seen in the cross‐validation), but overall, was neutral and not significant. Similarly, the models showed that A/H1N1 activity was not correlated with concurrent B activity.

The estimated effects of weather variables for A/H3N2 and influenza B, however, shifted toward the null, when adjusting for co‐circulating (sub)types (Figure S2 G‐L for A/H3N2 and M‐R for influenza B). In particular, for both viruses, the association with temperature, originally marginally negative for A/H3N2 and positive for B, was much closer to the null. The mean OR, averaged over 30 models, was 0.95 (vs 0.82 without the adjustment) for A/H3N2 and 1.08 (vs 1.19 without the adjustment) for influenza B.

Interestingly, the models consistently identified a strong negative association between A/H3N2 and concurrent B activity (Figure 3L, R). Each standard deviation (SD) increase in concurrent B activity was associated with a decreased OR of 0.44 [0.32, 0.60] (*P* < 1e‐6 for all 3 models) for A/H3N2 activity during 2007‐2015; conversely, each SD increase in concurrent A/H3N2 activity was associated with a decreased OR of 0.75 [0.58, 0.97] (*P* < .05 for all 3 models) for B activity during the same study period. In addition, this mutually negative association between A/H3N2 and B was evident in all cross‐validation tests (Figure 3L, R).

**Figure 4:**
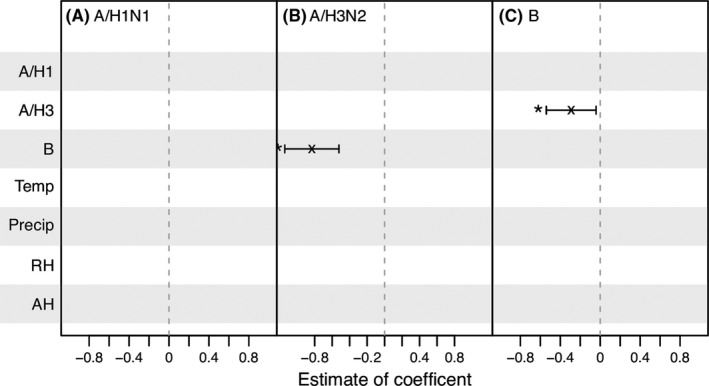
Best‐fit models for influenza epidemics in Entebbe during 2007‐2015. The variables tested are shown on the *y*‐axis. Coefficient estimates are shown for variables included in the best‐fit models (ie, the ones with the lowest BIC); the vertical segments show the 95% confidence intervals; “x”s denote the mean and “*”s indicate predictors significant at the 5% level. For instance, for A/H1N1 (1st column), no vertical segments appear, indicating the best‐fit model for A/H1N1 includes none of the tested variables; for A/H3N2 (2nd column), only 1 vertical segment, corresponding to concurrent B activity (labeled on the *y*‐axis), appears, indicating among the tested variables concurrent B activity is the only variable included in the best‐fit model

We then tested all possible combinations of weather variables and co‐circulating (sub)types as model predictors and pooled all models to identify the best‐fit model for each (sub)type during 2007‐2015, based on Bayesian information criterion (BIC). Model fits are shown in Figure S3. As shown in Figure 4, none of the 4 weather variables were included in the best‐fit models. However, a negative association between influenza A/H3N2 and B activity was identified in the best‐fit models for both viruses (Figure 4B, C). The estimated associations between the 2 viruses in the best‐fit models, where the opponent virus was the only additional predictor along with the lag‐1 and seasonality terms, were similar to the models with both co‐circulating (sub)types and weather variables (OR = 0.44 [0.32, 0.59], *P* = 1.4e‐7 for A/H3N2 v. B; and OR = 0.75 [0.58, 0.96], *P* = .022 for B v. A/H3N2).

### 3.3 Partial statistical analysis for Entebbe and Kampala

**Figure 5:**
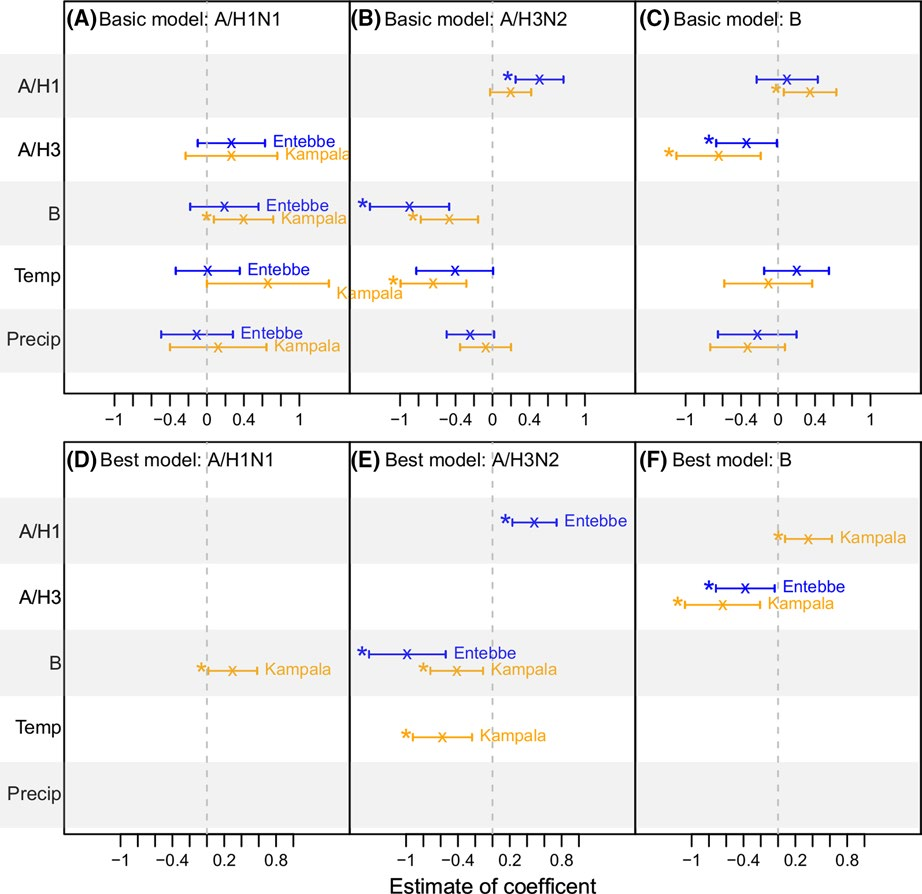
Comparison of estimated associations with weather variables and inter‐(sub)type interactions in Entebbe and Kampala. The upper panel (A‐C) shows estimates by the basic models specified in Equation (2), and the lower panel (D‐F) shows estimates by the best‐fit models (ie, the ones with the lowest BIC) among all tested models. The predictors are labeled along the *y*‐axis; the vertical segments show the 95% confidence intervals for each predictor for Entebbe (in blue) and Kampala (in orange); “x”s denote the mean and “*”s indicate predictors significant at the 5% level

To verify the relationships between influenza activity with weather conditions and co‐circulating (sub)types identified for Entebbe, we performed a similar statistical analysis for Kampala. However, due to a lack of data for humidity in Kampala, in this comparison, for both cities the models only included temperature and precipitation. Model fits for both cities are shown in Figure S4. Figure 5 shows the model coefficients estimated by the basic models with all variables included (A‐C) and the best‐fit models (D‐F). With a shorter model period (July 2008‐December 2014 excluding the 2009 pandemic) and only a subset of weather variables, the negative correlation between A/H3N2 and B activity, identified in the above full analysis (3.2.2), remained the most significant relationship in the models for both cities. This negative association was identified in both the basic models (Figure 5B, C) and the best‐fit models (Figure 5E, F). In addition, a mutually positive correlation between A/H1N1 and B activity was identified; however, this relationship was only significant for Kampala (Figure 5A, C, D, and F). For A/H3N2, concurrent A/H1N1 activity was identified as a positive correlate (*P* = .00012 for Entebbe and 0.085 for Kampala; Figure 5B); however, this positive correlation was not mutually identified in the A/H1N1 models (*P* = .15 for Entebbe and 0.29 for Kampala; Figure 5A). For the 2 weather variables examined, lower temperature was associated with higher A/H3N2 for both cities (*P* = .054 for Entebbe and *P* = .00042 for Kampala; Figure 5B). These estimated associations were in general in agreement across years as shown in the leave‐one‐out cross‐validation (Figure S5).

## 4 DISCUSSION

Using laboratory‐confirmed influenza surveillance data for 2 major Ugandan cities, we have examined the key weather variables and viral interactions shaping the epidemic dynamics of influenza in these tropical populations. For Entebbe over 2007‐2015, among all variables, intertype viral interactions were the most consistent and strongest predictors for influenza A/H3N2 and B epidemics: A/H3N2 activity was negatively associated with concurrent B activity and *vice versa*. After adjusting for co‐circulating influenza viruses, our models showed that epidemics of influenza A/H1N1 were significantly associated with lower precipitation and marginally associated with lower temperature, whereas A/H3N2 and B epidemics were not associated with either temperature or any of the 3 humidity variables (ie, precipitation, RH, or AH). The mutually negative association between A/H3N2 and B was also consistently identified for both Kampala and Entebbe over a shorter study period (July 2008‐December 2014).

Previous studies have reported inconsistent findings regarding meteorological effects on influenza transmission in the tropics, in particular, for humidity.^5^, ^7^, ^13^, ^14^, ^15^, ^16^, ^17^ These studies typically made inference based on epidemic time series for all influenza viruses combined, without distinguishing influenza (sub)type. Here, we found that the impact of weather conditions could vary by influenza (sub)type. While precipitation was identified as a significant correlate of A/H1N1 epidemics in Entebbe during 2007‐2015, the associations with activity of A/H3N2, B, or all influenza viruses combined were neutral. In addition, we showed in the leave‐one‐out cross‐validation that the correlations of influenza activity and weather variables also varied by study period. Given that circulating influenza viruses vary by location and year, the differing effects of weather conditions by influenza (sub)type and inter‐(sub)type interactions may in part explain the inconsistencies in the literature. Indeed, we showed that, with the adjustment for inter‐(sub)type interactions in the models, weak associations with weather variables (eg, temperature and A/H3N2 or, temperature and B in Entebbe) were modified toward the null while robust associations (eg, temperature and A/H1N1 in Entebbe) stayed unchanged. Future studies therefore should account for such interactions when assessing the impact of weather variables, in particular, for regions with concurrent circulation of multiple influenza (sub)types.

Influenza viruses mutate continuously to escape prior immunity and thus are able to cause recurrent epidemics. Nevertheless, prior infection may confer partial, or cross, immunity to future infection and lead to competing interactions among influenza viruses. Animal experiments and serological surveys have shown that cross‐immunity exists, not only in influenza strains of the same subtype but also across subtypes^27^, ^28^, ^29^, ^30^, ^31^, ^32^, ^33^, ^34^, ^35^ and types.^36^, ^37^, ^38^ Here, we showed that interactions among the 3 influenza (sub)types are a key determinant of the epidemic dynamics of individual (sub)types. In particular, a strong mutual negative association between A/H3N2 and B activity was consistently identified across years and the 2 Ugandan cities. This finding provides support for the impact of cross‐immunity at the population level. Further, as influenza vaccination coverage in the 2 studied Ugandan cities was near zero,^9^ the inter‐(sub)type interactions reported here likely reflect the impact of cross‐immunity conferred via natural infections, as opposed to vaccination.

Due to sparse observations of influenza infection and a lack of long‐term disease surveillance, we used monthly data for the analyses. This coarse time resolution likely limited our ability to identify the impact of weather variables, which tend to act acutely (eg, lower AH ~2 weeks prior to the epidemic onset^4^), and in part explains the null findings for relative and absolute humidity. This limitation stresses the need for enhanced surveillance and research on influenza in tropical regions,^39^ in particular, in Africa. In addition, due to a lack of publically available weather data, we were only able to conduct a partial comparison between the 2 Ugandan cities. Findings from this partial comparison were not entirely consistent between the 2 cities (except for the negative association between A/H3N2 and B). This inconsistency could stem from a lack of statistical power due to low signal/noise ratios, or location‐specific factors (eg, population immunity) that were not accounted for in the models. Further investigations into these issues are warranted.

Despite the limited disease data, we have shown differing correlations with weather variables for different influenza (sub)types as well as key viral interactions among co‐circulating (sub)types at the population level in 2 tropical cities. Future work using data with better temporal resolutions and from more tropical locations hopefully will reveal a more comprehensive picture of the dynamics of influenza epidemics in tropical climates.

## COMPETING INTERESTS

JS discloses partial ownership of SK analytics; WY discloses consultation for SK analytics. No other disclosures are reported.
