# Supplementary material for: Dynamics of influenza in tropical Africa: Temperature, humidity, and co‐circulating (sub)types
Source: Influenza Other Respir Viruses. 2018 Apr 17;12(4):446–56. doi: 10.1111/irv.12556 (PMC6005592; doi:10.1111/irv.12556)
Supplement: Supplementary file 1 [file IRV-12-446-s001.docx]

**Supplementary material**

**Dynamics of Influenza in Tropical Africa: Temperature, Humidity, and Co-circulating (sub)types**

Wan Yang, Matthew J. Cummings, Barnabas Bakamutumaho, John Kayiwa, Nicholas Owor, Barbara Namagambo, Timothy Byaruhanga, Julius J. Lutwama, Max R. O’Donnell, Jeffrey Shaman

**Fig S1**. Weather conditions in Entebbe and Kampala.

**Fig S2.** Comparison of estimated associations of influenza activity with weather variables, by models without adjusting for co-circulating (sub)types (in blue) and those with the adjustment (in orange). Model settings are the same as in Figs 2 and 3. The values on the top show the mean for the coefficient, averaged over all models, and the 95% confidence interval of the mean estimate.

**Fig S3.** Model fits from the best-fit models for Entebbe in the full analysis. ‘x’s are observed monthly viral positive rates; black lines are model estimates and grey areas surrounding the black lines show the 95% confidence interval.

**Fig S4**. Model fits from the best-fit models for Entebbe and Kampala in the partial analysis. ‘x’s are observed monthly viral positive rates; black lines are model estimates and grey areas surrounding the black lines show the 95% confidence interval.

**Fig S5** Comparison of estimated associations with weather variables and inter-(sub)type interactions in Entebbe and Kampala. Coefficient estimates are based on the basic models specified in Eqn 2 in the leave-one-out cross-validation and over the entire study period (July 2008 – Dec 2014). The year left out in the cross-validation is shown on the y-axis (e.g., ‘-2008’ indicates data for Year 2008 were excluded, and ‘-none’ indicates data for the entire study period were included). The associations with precipitation (Precip) and temperature (Temp) are shown in columns 1 and 2, and (sub)type interactions are shown in columns 3 and 4; the vertical segments show the 95% confidence intervals for each variable for Entebbe (in blue) and Kampala (in orange); ‘x’s denote the mean and ‘*’s indicate variables significant at the 5% level. The values on the top of each plot show the mean for the coefficient, averaged over all models, and the 95% confidence interval of the mean estimate.
